# Cranioplasty After Severe Traumatic Brain Injury: Effects of Trauma and Patient Recovery on Cranioplasty Outcome

**DOI:** 10.3389/fneur.2018.00223

**Published:** 2018-04-09

**Authors:** Jussi P. Posti, Matias Yli-Olli, Lauri Heiskanen, Kalle M. J. Aitasalo, Jaakko Rinne, Ville Vuorinen, Willy Serlo, Olli Tenovuo, Pekka K. Vallittu, Jaakko M. Piitulainen

**Affiliations:** ^1^Division of Clinical Neurosciences, Department of Neurosurgery, Turku University Hospital, Turku, Finland; ^2^Division of Clinical Neurosciences, Turku Brain Injury Centre, Turku University Hospital, Turku, Finland; ^3^Department of Neurology, University of Turku, Turku, Finland; ^4^Department of Biomaterials Science and Turku Clinical Biomaterials Centre––TCBC, Institute of Dentistry, University of Turku, Turku, Finland; ^5^Department of Otorhinolaryngology––Head and Neck Surgery, Division of Surgery and Cancer Diseases, Turku University Hospital, Turku, Finland; ^6^Department of Children and Adolescents, Oulu University Hospital, Oulu, Finland; ^7^MRC Oulu, PEDEGO Research Center, Oulu University, Oulu, Finland; ^8^City of Turku Welfare Division, Turku, Finland

**Keywords:** cranioplasty, decompressive craniectomy, imaging, outcome, severe traumatic brain injury

## Abstract

**Background:**

In patients with severe traumatic brain injury (sTBI) treated with decompressive craniectomy (DC), factors affecting the success of later cranioplasty are poorly known.

**Objective:**

We sought to investigate if injury- and treatment-related factors, and state of recovery could predict the risk of major complications in cranioplasty requiring implant removal, and how these complications affect the outcome.

**Methods:**

A retrospective cohort of 40 patients with DC following sTBI and subsequent cranioplasty was studied. Non-injury-related factors were compared with a reference population of 115 patients with DC due to other conditions.

**Results:**

Outcome assessed 1 day before cranioplasty did not predict major complications leading to implant removal. Successful cranioplasty was associated with better outcome, whereas a major complication attenuates patient recovery: in patients with favorable outcome assessed 1 year after cranioplasty, major complication rate was 7%, while in patients with unfavorable outcome the rate was 42% (*p* = 0.003). Of patients with traumatic subarachnoid hemorrhage (tSAH) on admission imaging 30% developed a major complication, while none of patients without tSAH had a major complication (*p* = 0.014). Other imaging findings, age, admission Glasgow Coma Scale, extracranial injuries, length of stay at intensive care unit, cranioplasty materials, and timing of cranioplasty were not associated with major complications.

**Conclusion:**

A successful cranioplasty after sTBI and DC predicts favorable outcome 1 year after cranioplasty, while stage of recovery before cranioplasty does not predict cranioplasty success or failure. tSAH on admission imaging is a major risk factor for a major complication leading to implant removal.

## Introduction

Decompressive craniectomy (DC) is a neurosurgical emergency procedure in which a large section of the skull is removed and the dura mater is opened. A large bone flap is left out in order to allow brain tissue to expand and thus to lower intracranial pressure (ICP) (1). Indications for DC are medically refractory elevated ICP due to severe traumatic brain injury (sTBI) and other causes of intractable brain swelling such as stroke, subarachnoid hemorrhage, and intracerebral hemorrhage (2).

Decompressive craniectomy leaves a bony skull defect. It exposes brain tissue to atmosphere pressure and disturbs physiological brain perfusion (3) and cerebrospinal fluid flow (4), and exposes to later neurological symptoms such as epileptic seizures (5). “Syndrome of the trephined” is a variable post-craniectomy condition that is commonly characterized by motor, cognitive, and language deficits, which often resolve after cranial reconstruction (6).

Cognitive and neurological deficits occur typically in patients with sTBI after DC (6) and due to the serious nature of the condition, patients with sTBI are also prone to develop surgical complications after cranioplasty. With present surgical methods and different implant materials, cranioplasty is associated with a high complication rate with the main complication being infection (7). However, cranioplasty is reported to improve cognitive performance and thus the procedure is important to patients who suffer from sequels of sTBI and DC (8). Therefore, we sought to investigate if injury- and treatment-related factors or state of recovery of the patient could predict the risk of major complications in cranioplasty procedure requiring implant removal, and how these complications affect the outcome.

## Materials and Methods

### Selection Criteria and Study Population

The medical records on all patients who had undergone cranioplasty at Turku University Hospital, Turku, Finland, from June 2002 through March 2015 were reviewed. The inclusion criteria for the primary patient cohort (patients with sTBI and DC) were (i) sTBI needing a DC due to refractory ICP and (ii) a subsequent cranioplasty, and (iii) available clinical, radiological, cranioplasty follow-up, and outcome data. The inclusion criteria for the reference population (patients with DC due to other reasons) were (i) a DC and a subsequent cranioplasty operation due to a malignant middle cerebral artery stroke, intracranial tumor, infection, spontaneous intracerebral hemorrhage, subarachnoid hemorrhage, and various other reasons treated at Turku University Hospital, Finland during the same time interval, and (ii) available clinical, radiological, and follow-up data. The patients were considered eligible for cranioplasty if they were conscious and being treated at least in long-term care facilities. All the patients were preoperatively evaluated by a surgeon and patients with signs of infection or scalp problems were not considered eligible for cranioplasty.

This is a retrospective registry study and the cohort includes patients whom were recruited in prospective clinical trials studying glass fiber-reinforced-bioactive glass composite (FRC-BG) implants (ClinicalTrials.gov identifiers NCT01874613 and NCT01202838). Both of study protocols of these studies were reviewed and approved by the Joint Commission on Ethics of Hospital District of Southwest Finland (§125/2008 and §118/2012). All of the patients provided their informed consents. The aims of the studies were to investigate functional and esthetic outcome and safety of cranioplasty using FRC-BG implants. For the current study, no ethics committee approval was needed, as this was a retrospective registry study. FRC-BG implants are currently in routine clinical use in Finland. The study was approved by Turku University Hospital, Turku. Finland. Turku University Hospital has departments of neurosurgery and head and neck surgery that have been responsible for craniofacial reconstructive surgery of people living in in Southwest Finland, Satakunta, and Åland Islands areas (combined population of 725,000) during the years 2002–2015.

### Data Collection

Clinical, radiological, and outcome data were collected, and a database was constructed using IBM SPSS Statistics version 23.0 (IBM Corporation, Armonk, NY, USA). The medical records were reviewed for the following clinical covariates for both of the populations: age, gender, diabetes, history of smoking, time interval between DC and cranioplasty, presenting diagnosis, material used for skull bone defect reconstruction, defect size, cranioplasty follow-up data, infection, and complications related to cranioplasty.

Cranioplasty follow-up data were determined at the following time points: 1 month, 6 months, and 12 months. The follow-up status was defined as normal when no wound healing problems or other complications were observed. Complications were defined as minor when conservative treatment or a minor local procedure was employed in case of wound problems and major when revision surgery and implant removal was required. The sizes of each defect were calculated from preoperative computed tomography (CT) images. A radiologist and a neurosurgeon (JPP) reviewed the intracranial findings on admission CT.

For patients with sTBI and DC the following clinical variables we additionally reviewed: level of consciousness with Glasgow Coma Scale (GCS) (9) at admission, presence or absence of pupillary reactions to light, CT findings upon admission, neurological outcome 1 day before the cranioplasty and 1 year after the cranioplasty as measured with Glasgow Outcome Scale (GOS) (10), concurrent hematoma evacuation with DC, subsequent extension of DC due to brain herniation, length of stay at the intensive care unit (ICU), and application of ventriculo-peritoneal shunt. GOS of 1–3 was classified unfavorable (dead or dependent on others) and 4–5 favorable (independent). The patients with DC and subsequent cranioplasty due to other reasons than TBI have undergone a different follow-up scheme and their initial clinical characteristics are recorded in a different manner. Due to this, GOS and GCS data are not available for this group.

The association of outcome and cranioplasty complications was further investigated using validated and established CRASH prediction model (11) that is being used for outcome prediction of patients with TBI. The clinical and radiological covariates included in the model were independently studied.

### Decompressive Hemicraniectomy

At the ICU of Turku University Hospital, the standard protocol for therapeutic management for ICP aims to maintain ICP <20 mmHg and cerebral perfusion pressure >60 mmHg by applying treatments in a stepwise scheme. All patients with GCS score <8 receive intraparenchymal ICP monitoring probe on a standard basis. If ICP remains >20 mmHg despite of maximal medical therapy and insertion of a possible ventriculostomy, a large unilateral frontotemporoparietal DC (hemicraniectomy) is performed based on neurosurgeon’s decision.

Decompressive craniectomy was classified as primary when the decompression was done due to refractory ICP or concomitantly with intracranial hematoma evacuation and secondary when a hematoma evacuation was performed initially, but ICP became refractory afterward necessitating DC.

### Cranioplasty Materials

Different materials have been used in cranioplasty, including frozen autologous bone, hydroxyapatite (HA) bone cement, *in situ* cured polymethyl methacrylate (PMMA) bone mass, prefabricated PMMA implant with bioactive glass (BG) particle coating, titanium (Ti) mesh or bulk Ti implant, polyetheretherketone (PEEK), polyethylene (PE), and FRC-BG implant (12, 13). For statistical analyses, HA, PMMA, PEEK, PE, FRC-BG, and Ti were categorized as implants and autologous bone flap as autograft. During the study period, frozen autologous bone flaps have been used when available, but in case of a fracture in an autologous bone flap or a contamination, synthetic materials have been utilized. The choice of synthetic material has been based on a clinical decision and sometimes on competitive tendering if there has been no clinical preference announced by a surgeon.

At our center, surgical wound drains are not routinely used. However, drains have been used *ad hoc* in cases of perforated custom-made PMMA and Ti mesh implants in order to prevent epidural hematoma if there has been a concern about scalp hemostasis.

Infection prophylaxis with intravenous antibiotics was given according to department-specific protocols (cefuroxime 3 g preoperatively and 1.5 g three times daily for 1–3 days after cranioplasty). Autologous bone flaps were stored at −80°C under sterile conditions until cranioplasty. Abdominal implantation was not used. On a routine basis, a microbiological sample was obtained before storing autologous bone flaps. If bacterial growth was detected, the bone flap was discarded.

### Statistical Analyses and Data Handling

Statistical analyses were performed using IBM SPSS Statistics version 23.0 and 24.0 (IBM Corporation, Armonk, NY, USA) and JMP Pro version 12.0 (SAS Institute North Carolina, 27513, USA).

Differences in complication rates between the study populations were studied using two-tailed χ^2^ test. Differences in complication rates between time intervals were studied using two-tailed Fisher’s exact test, logistic regression, and Omnibus tests of model coefficients. Differences in GOS progression between 1 day before cranioplasty and 1 year after cranioplasty was studied Mann–Whitney U test (two-sample test) between patients with successful cranioplasty and patients with major complication leading to implant removal. The changes in GOS scores were not normally distributed. Differences in complication rates between different outcome groups 1 year after cranioplasty were studied with two-tailed Fisher’s exact test and logistic regression and Omnibus tests of model coefficients. Differences between complication rates in different time intervals between DC and cranioplasty were studied with Fisher’s exact test and logistic regression was further used to assess if time interval could be used to predict incidence of complications. Differences in complication rates between time of ICU stay were assessed using two-tailed Fisher’s exact test. The correlation of GOS scores day before and 1 year after cranioplasty with cranioplasty success and failure were studied with logistic regression. The effects of CRASH covariates on outcome and cranioplasty complications were studied with two-tailed Fisher’s exact test and logistic regression. A *p* < 0.05 was considered significant.

## Results

Forty patients with sTBI requiring a DC and a subsequent cranioplasty procedure were considered eligible for this retrospective study. The reference population consisted of 115 patients with DC and a subsequent cranioplasty operation due to infections (*n* = 41, 36%), intracranial tumor (*n* = 31, 27%), malignant middle cerebral artery stroke (*n* = 15, 13%), spontaneous intracerebral hemorrhage (*n* = 13, 11%), subarachnoid hemorrhage (*n* = 8, 7%) or other reasons (*n* = 7, 6%).

The mean age of the patients with TBI and DC at the time of cranioplasty was 36 ± 16 years and the majority were male (78%). In patients with DC due to other reasons, the mean age was 46 ± 17 years and the majority were male (62%). Demographic data are presented in Table 1.

**Table 1:** Descriptive statistics.

	Patients with TBI and DC			Patients with DC due to other reasons		
	*n* (%)	Mean	SD	*n* (%)	Mean	SD
*N*	40			115		
Age (years)		36	16.5		46	17.8
Smoking	12 (30)			25 (22)		
Gender						
Male	31 (77.5)			71 (61.7)		
Female	9 (22.5)			44 (38.3)		
Cranioplasty material						
Autograft	17 (42.5)			27 (23.5)		
Ti	8 (20.0)			24 (20.9)		
HA	6 (15.0)			25 (21.7)		
FRC-BG	5 (12.5)			18 (15.7)		
PMMA	2 (5.0)			8 (7.0)		
PEEK	1 (2.5)			3 (2.6)		
PE	1 (2.5)			10 (8.7)		
Skull defect size (cm^2^)		106.4	75.0		87.4	72.5
CT findings						
Midline shift	31 (77.5)					
SDH	27 (67.5)					
tSAH	23 (57.5)					
Contusion	24 (60.0)					
Petechial hemorrhage	17 (42.5)					
Obliteration of III ventricle and cisterns	17 (42.5)					
EDH	9 (22.5)					
GCS (3–15)		7.4	4.8			
GOS a day before cranioplasty (1–5)		3.5	0.8			
GOS 1 year after cranioplasty (1–5)		3.9	0.9			
Time between DC and cranioplasty (days)		330	250.4			
ICU stay (days)		13.7	7.8			
Primary DC	32 (80.0)					
Secondary DC	8 (20.0)					
Concurrent DC and hematoma evacuation	28 (70.0)					
Subsequent enlargement of DC	12 (30.0)					

*Ti, commercially pure titanium (mesh or bulk), HA, hydroxyapatite; FRC-BG, glass fiber-reinforced-bioactive glass composite (S53P4); PMMA, polymethyl methacrylate; PEEK, polyetheretherketone; PE, polyethylene; SDH subdural hematoma; tSAH, traumatic subarachnoid hemorrhage; EDH, epidural hematoma; GCS, Glascow Coma Scale; GOS, Glasgow Outcome Scale; DC, decompressive craniectomy*.

Of patients with DC and TBI, 65% (*n* = 26/40) underwent a concomitant intracranial hematoma evacuation with DC and 15% (*n* = 6/40) did not have a hematoma requiring evacuation at the time of DC (solitary diffuse injury); thus, 80% (*n* = 32/40) of patients underwent a primary DC and 20% (8/40) underwent a secondary DC. There were no open calvarial fractures in the study cohort.

### Major Cranioplasty Complications in Study Populations

In patients with sTBI and DC, the overall rate for a major complications requiring implant removal after cranioplasty was 18% (*n* = 7/40) and rate for minor complications not leading to implant removal was 18% (*n* = 7/40). Thus, the overall complication rate was 35% (*n* = 14/40) in patients with sTBI and DC.

In patients with DC due to other reasons overall rate for a major complications requiring implant removal after cranioplasty was 24% (*n* = 28/115), giving a major complication rate for both cohorts of 23% (*n* = 35/155). Between these two cohorts, no difference was observed in major complication rates (χ^2^ = 0.796; *p* = 0.372).

### Pre-Existing Conditions

In this study, age, diabetes, and history of smoking did not have a significant effect on the major complication rate in patients with TBI and DC or in patients with DC due to other reasons.

### Cranioplasty Materials and Skull Defect Size

In patients with TBI and DC, major complication rate in autograft group was 24% (*n* = 4/17). When complication rates between autograft and implant groups were studied, no difference was detected (*p* = 0.432). In patients with DC due to other reasons, major complication rate in autograft group was 26% (*n* = 7/27). When complication rates between autograft and implant groups were studied, no difference was detected (*p* = 0.560). The cranioplasty material results are presented in Table 2.

**Table 2:** Cranioplasty materials and major complications leading to implant removal.

	Patients with TBI and DC			Patients with DC due to other reasons		
	Major complication *n*	Major complication %	Total	Major complication *n*	Major complication %	Total
Autograft	4	23.5	17	7	25.9	27
Ti	1	12.5	8	3	12.5	24
HA	1	16.7	6	7	28.0	25
FRC-BG	1	20.0	5	6	33.3	18
PMMA	0	0	2	2	25.0	8
PEEK	0	0	1	0	0	3
PE	0	0	1	3	30	10

*DC, decompressive craniectomy; Ti, commercially pure titanium (mesh or bulk), HA, hydroxyapatite; FRC-BG, glass fiber-reinforced-bioactive glass composite (S53P4); PMMA, polymethyl methacrylate; PEEK, polyetheretherketone; PE, polyethylene*.

There was no correlation with skull defect size and major complications leading to implant removal.

### Time Interval Between Decompressive Craniectomy and Cranioplasty

In both patients with TBI and DC and patients with DC due to other reasons, there was no correlation between time interval between DC and cranioplasty and major complication rate leading to implant removal. Only groups 3–6 months and 6–12 months are compared due to low frequencies in the other groups (Table 3, highlighted).

**Table 3:** Time interval between decompressive craniectomy and cranioplasty and comparison of major complications.

	Patients with TBI and DC				Patients with DC due to other reasons			
	Major complication *n*	Major complication %	Total	*p*-Value	Major complication n	Major complication %	Total	*p*-Value
0–3 months	0	0	2		4	12.1	33	
3–6 months	2	22.2	9	0.999^a^	4	33.3	12	0.058^a^
6–12 months	5	27.8	18		7	19.4	36	
>12 months	0	0	11		13	38.2	34	

*The p-values are from Fisher’s exact Test*.

*^a^Zero values cannot be assessed with Fisher’s exact test and thus only groups of 3–6 months and 6–12 months are compared in patient both groups (highlighted)*.

However, when a linear regression model was utilized, a time of cranioplasty can be significantly used to predict the incidence of complications (*p* = 0.007) and the regression variables significantly differ from zero (*p* = 0.032) in patients with DC due to other reasons.

### Traumatic Brain Injury, Treatment, and Stay at the ICU

All patients were treated at the ICU. The reasons for staying at the ICU were ICP problems, low level of consciousness, and dependency on mechanical ventilation. There was no correlation between the time of stay at the ICU and major complications.

There was no difference in major complication rates leading to implant removal in patients with a concomitant hematoma evacuation during DC and in patients with DC after hematoma evacuation. Furthermore, no difference between major complication rates between patients requiring an enlargement of the initial DC due to herniating brain and in patients without a need for enlargement was observed.

### Outcome After Severe Traumatic Brain Injury

When the GOS was assessed 1 day before cranioplasty and compared with GOS 1 year after cranioplasty, there was a significant improvement of GOS in patients with a successful cranioplasty as compared with patients with major complication leading to implant removal (*p* = 0.015).

In patients with favorable outcome (GOS 4–5), 1 year after cranioplasty, major complication rate leading to implant removal was 7% (*n* = 2/28) and in patients with unfavorable outcome (GOS 1–3) the major complication rate was 42% (*n* = 5/12) with a significant difference (*p* = 0.003). However, when differences in GOS scores on day before cranioplasty were analyzed in patients with successful cranioplasty and patients with implant removal, no differences were detected (*p* = 0.46).

Of CRASH covariates, the following findings were associated with unfavorable outcome: lost pupillary reactions to light (*p* = 0.031), petechial hemorrhages on CT (*p* = 0.001), obliteration of the third ventricle or basal cisterns on CT (*p* = 0.013), and traumatic subarachnoid hemorrhage (tSAH) on CT (*p* < 0.001). Other clinical and imaging covariates were not associated with GOS.

### Factors Correlated With Outcome of Cranioplasty

When studied in a logistic regression model, the GOS 1 year after cranioplasty was significantly correlated with cranioplasty outcome (odds ratios from logistic regression model 12.11 and 0.083, for normal healing and major complication leading to implant removal, respectively, *p* = 0.022). In a similar regression model, GOS score day before cranioplasty could not predict major cranioplasty complications (*p* = 0.097). The associations of GOS scores with major cranioplasty complications are demonstrated in Table 4. No cases of death occurred during the follow-up period.

**Table 4:** The correlation of Glasgow Outcome Scale score (A) 1 day before cranioplasty and (B) 1 year after cranioplasty with cranioplasty success (normal healing) and cranioplasty failure (major complication leading to implant removal) as studied with a logistic regression model.

A					Cranioplasty success			Cranioplasty failure		
	GOS 1 day before cranioplasty	Major complication *n*	Major complication %	Total	*B*	Exp(*B*)	*p*	*B*	Exp(*B*)	*p*
Unfavorable	GOS 2	2	33.3	6	–1.78	0.17	0.097	1.78	5.93	0.097
	GOS 3	2	15.4	13						
Favorable	GOS 4	3	17.6	17						
	GOS 5	1	25	4						

**B**					**Cranioplasty success**			**Cranioplasty failure**		
	**GOS 1 year after cranioplasty**	**Major complication *n***	**Major complication %**	**Total**	***B***	**Exp(*B*)**	***p*-Value**	***B***	**Exp(*B*)**	***p*-Value**

Unfavorable	GOS 2	3	100	3	2.49	12.11	**0.022**	–2.49	0.083	**0.022**
	GOS 3	2	22.2	9						
Favorable	GOS 4	2	10.5	19						
	GOS 5	0	0	9						

*GOS, Glasgow Outcome Scale; B, logistic regression coefficient; Exp(*B*), odds ratio from logistic regression; *p, p*-value*.

As GOS 1 year after cranioplasty was significantly correlated with operation outcome, the association of covariates included in CRASH model and GOS 1 year after cranioplasty were then investigated.

The correlation of CRASH model covariates and major cranioplasty complications leading to implant removal were studied. Of patients with tSAH on CT, 30% (*n* = 7/23) developed a major complication, while none of patients without tSAH (*n* = 0/17) had a major complication (*p* = 0.014). When studied in a logistic regression model, tSAH significantly predicted a major complication (*p* = 0.003). Other CRASH covariates that were correlated with unfavorable outcome (see factors correlated with outcome of cranioplasty) did not predict a major cranioplasty complication.

## Discussion

There are two main findings in this study. First, a successful cranioplasty after sTBI and DC predicts favorable outcome 1 year after cranioplasty, whereas patient outcome as assessed before cranioplasty does not predict cranioplasty success or failure. In other words, the rate of major cranioplasty complications is similar regardless of the neurological state of a patient at the time of cranioplasty. Second, tSAH detected on admission head CT significantly predicts the risk for subsequent major cranioplasty complications, while other injury-related clinical and radiological variables do not.

In order to shed more light on complications related to cranioplasty after TBI, a reference population with other indications for DC was analyzed concomitantly. There were no differences in the number of cranioplasty complications between the patient groups. Clinical variables that have earlier been demonstrated to have effect on cranioplasty complications, such as age (14) and diabetes (15), did not show significant effect on cranioplasty success in either of the populations in this study. Smoking has been associated with surgical site infection (16), but showed no significance in this study. These negative findings in the current study may be related to the small study cohort size. Different cranioplasty materials were not associated with the rate of cranioplasty complications.

We found that there was a significant improvement of outcome in patients with successful cranioplasty as compared with patients with major complication leading to implant removal. The findings suggest that patients with history of sTBI and DC may generally be good candidates for cranioplasty despite of a level of recovery and when successful, cranioplasty significantly improves patient outcome. Another possible explanation is that those patients who still have a good recovery potential at the time of cranioplasty are less likely to develop complications. Because the level of GOS before cranioplasty did not predict complications, we feel that either a successful cranioplasty is enhancing the recovery or that a cranioplasty complication increases the risk for poor recovery, or both.

Cranioplasty restores the contour of the skull, restores physiological intracranial dynamics, and protects the brain. Thus, it is plausible the reconstructed skull after sTBI and subsequent DC improves cerebral perfusion in different lobes (8). It has been shown that common post-craniectomy symptoms including cognitive, language, and motor decline are often partially reversed after cranioplasty (6, 8, 17, 18). Additionally, cranioplasty is shown to ameliorate and accelerate cognitive profile improvement in a non-specific manner if performed in early phase (19). Thus, a beneficial effect of cranioplasty on improved neurological outcome appears as a meaningful explanation in the current study.

Many clinical and radiological covariates are found to correlate with outcome of TBI (11, 20–23). In this study, we found that lost pupillary light reactions and signs of diffuse brain injury such as petechial hemorrhages and tSAH were associated with poor neurological outcome. Based on this, we sought to investigate, which of these covariates correlate with cranioplasty failure and implant removal. We found that tSAH upon admission was a significant predictor of a major cranioplasty complication. This finding may be interpreted so that diffuse TBI as depicted partially by tSAH is associated with compromised neurological outcome as time passes (11, 22, 23) and thus predisposes patients to higher incidence of complications.

We recognize that there are limitations in this study. First, the study cohort is relatively small. During the study period (2002–2015), DC treatment protocol for malignant middle cerebral artery stroke was already mainly established, while DC for sTBI was still undertaken based on individual decision by a neurosurgeon––usually on call. This may also have caused a relative selection bias as compared with a cohort recruited today. Second, due to the retrospective setting, there might be inaccuracies in functional outcome grading. However, at our institution, all patients with sTBI and DC are carefully evaluated before and after cranioplasty. If the patient is considered eligible for skull reconstruction, he undergoes another neurological examination and an interview about his current condition. Relatives and/or treating health care professionals are usually interviewed in parallel. Cranioplasty patients visit neurosurgical outpatient clinic 3–6 months after cranioplasty and they visit brain trauma outpatient clinic for rehabilitation evaluation subsequently. Every patient with sTBI is admitted rehabilitation at our institution and detailed follow-up notes are available. In case a patient has not moved away from the hospital district area, all the postoperative complications are treated at our institution. In such case, elaborate notes of patient’s neurological examination and overall condition are available. Based on these circumstances, the five-step grading of GOS can be considered to be adequate in this study. Regarding the decision to reconstruct the skull after DC the patient selection bias can be considered minor, because all meaningfully responsive patients are admitted to cranioplasty on a regular basis at our institution.

## Conclusion

In conclusion, the current findings show that a successful cranioplasty after sTBI and DC predicts favorable outcome one year after cranioplasty, while patient outcome as assessed before cranioplasty does not predict cranioplasty success or failure. Patients with tSAH on admission CT are prone to experience major cranioplasty complications, but on the other hand, initial state of consciousness and longer need for ICU treatment are not associated with cranioplasty failure. Patients who are initially unconscious, and have even lost their pupillary reactions, and have sustained major extracranial injuries appear adequate candidates for cranioplasty. These patients may show ameliorated outcome after cranioplasty. The rate of major cranioplasty complications is similar regardless of the neurological state of a patient at the time of cranioplasty.

## Ethics Statement

This is a retrospective registry study and the cohort includes patients whom were recruited in prospective clinical trials studying glass fiber-reinforced-bioactive glass composite (FRC-BG) implants (ClinicalTrials.gov identifiers NCT01874613 and NCT01202838). Both of study protocols of these studies were reviewed and approved by the Joint Commission on Ethics of Hospital District of Southwest Finland (Protocol no. 167;125/2008 and Protocol no. 167;118/2012). All of the patients provided their informed consents. The aims of the studies were to investigate functional and aesthetic outcome and safety of cranioplasty using FRC-BG implants. For the current study, no ethics committee approval was needed, as this was a retrospective registry study. FRC-BG implants are currently in routine clinical use in Finland. The study was approved by Turku University Hospital, Turku. Finland.

## Author Contributions

JPP, MYO and JMP conceived and designed the study and collected the data. JPP, JMP, KMJA, VV, WS and PKV designed the data collection for the cranial implant registry at Turku University Hospital. LH conducted the statistical analyses with a contribution from JPP. JPP drafted the manuscript with critical contributions from MYO and LH. All authors substantially contributed to the revision of the manuscript. JPP takes the responsibility for the paper as whole.

## Conflict of Interest Statement

JPP, MY-O, LH, KMJA, JR, VV, WS, OT and JMP have no financial disclosures. PKV is a Member of the Board and shareholder of Skulle Implants Corp. The reviewer AL and handling Editor declared their shared affiliation.
